# The prognostic value of the Tau protein serum level in metastatic breast cancer patients and its correlation with brain metastases

**DOI:** 10.1186/s12885-019-5287-z

**Published:** 2019-01-30

**Authors:** Amélie Darlix, Christophe Hirtz, Simon Thezenas, Aleksandra Maceski, Audrey Gabelle, Evelyne Lopez-Crapez, Hélène De Forges, Nelly Firmin, Séverine Guiu, William Jacot, Sylvain Lehmann

**Affiliations:** 1 Department of Medical Oncology, Institut du Cancer de Montpellier, University of Montpellier, 208 Avenue des Apothicaires, 34298 Montpellier, France; 2 Laboratoire de Biochimie et Protéomique Clinique, University of Montpellier, Institute of Regenerative Medicine – Biotherapy IRMB, CHU Montpellier, INSERM, 80 Avenue Augustin Fliche, 34295 Montpellier, France; 3 Biometrics Unit, Institut du Cancer de Montpellier, University of Montpellier, 208 Avenue des Apothicaires, 34298 Montpellier, France; 4 Memory Resources and Research Center, University of Montpellier MUSE, CHU Montpellier, 80 avenue Augustin Fliche, 34295 Montpellier, France; 5 Translational Research Unit, Institut du Cancer de Montpellier, University of Montpellier, 208 Avenue des Apothicaires, 34298 Montpellier, France; 6 Clinical Research Unit, Institut Régional du Cancer de Montpellier, University of Montpellier, 208 Avenue des Apothicaires, 34298 Montpellier, France

**Keywords:** Tau protein, Breast cancer, Brain metastases, Tumor markers, Predictive factors, Prognostic factors

## Abstract

**Background:**

Metastatic breast cancer (MBC) prognosis is variable, depending on several clinical and biological factors. A better prediction of a patient’s outcome could allow for a more accurate choice of treatments. The role of serum biomarkers in predicting outcome remains unclear in this setting. Tau, a microtubule-associated protein, is a neuronal marker that is also expressed in normal breast epithelial cells and cancer cells. Its tissue expression is associated with prognosis in MBC. However, the prognostic value of Tau serum levels in these patients is unknown. We aimed at evaluating the prognostic value of Tau (and other classical biomarkers) in MBC patients, and to assess its association with the presence of brain metastases (BM).

**Methods:**

244 MBC patients treated at our institution (2007–2015) were retrospectively selected. The usual MBC clinical and pathological variables were collected, altogether with CA15–3, CEA and HER2 extra-cellular domain (ECD) serum levels. Tau serum levels were measured with a novel immunoassay (digital ELISA) using Single Molecule Array (Simoa) technology. Overall survival (OS) was estimated with the Kaplan-Meier method. To investigate prognostic factors, a multivariate analysis was performed. Cut-offs were set using the Youden index method associated with receiver-operating characteristics (ROC) curves to evaluate the accuracy of biomarkers to identify patients with BM.

**Results:**

With a median follow-up of 40.8 months, median OS was 15.5 months (95%CI 12.4–20.2). Elevated serum levels of Tau were independently associated with a poor outcome in the whole population as well as in patients with (*n* = 86) and without BM (*n* = 158). Median serum Tau levels tended to be higher in patients with BM (*p* = 0.23). In univariate analysis, patients with BM had an increased risk of serum Tau > 3.17 pg/mL (OR = 2.2, *p* = 0.049). In multivariate analysis, high values of Tau (OR = 3.98, *p* = 0.034) accurately identified patients with BM in our cohort.

**Conclusions:**

Tau is a new biomarker of interest in MBC. Its serum level could represent an independent prognostic factor in these patients (both with and without BM). It also seems to be associated with the presence of BM. A validation of these results in an independent set of MBC patients is necessary to confirm these findings.

**Electronic supplementary material:**

The online version of this article (10.1186/s12885-019-5287-z) contains supplementary material, which is available to authorized users.

## Background

The survival of metastatic breast cancer (MBC) patients has improved over the past decades with the use of new therapeutic agents [1]. However, the outcome remains poor, with a median overall survival (OS) of 21 to 30 months [1, 2]. MBC survival ranges from a few months to decades depending on various factors [4, 5]. Given this great variability in terms of outcome, probably reflecting the biological heterogeneity among MBC [3], a better evaluation of a given patient’s prognostic factors could allow a more accurate choice of therapeutic strategy.

To date, several clinical and biological prognostic factors have been reported, including patients’ characteristics (age and performance status), previous medical history (prior chemotherapy [CT] for MBC treatment) and disease extension (number and location of metastatic sites) [4–6]. Tumor biology (including hormone receptors [HR] and Human Epidermal growth factor Receptor 2 [HER2] expression) is also a cornerstone of patients’ prognosis. HR-positive tumors have been associated with a better outcome, and with response to hormone therapy [4, 7, 8]. Regarding the HER2 status, series published in the pre-trastuzumab era have demonstrated a negative prognostic value of HER2 amplification in MBC [9]. Since trastuzumab introduction, however, this effect has been reversed and HER2 amplification has been associated with a better outcome in recent series [5, 10]. High Lactate Dehydrogenase (LDH) and low albumin serum levels have also been reported as poor prognostic factors in MBC in several studies [11–13].

The role of serum tumor markers in predicting outcome remains unclear in patients with MBC. High Cancer Antigen 15–3 (CA 15–3) and Carcinoembryonic Antigen (CEA) serum levels seem to be associated with a poorer survival [14–17]. The prognostic value of serum HER2 extra-cellular domain (ECD) has been shown in MBC patients, whatever the HER2 status of the primary tumor, high serum HER2 ECD levels being associated with a poor outcome [17–19]. Considering the recent interest in phenotypic changes and tumor heterogeneity during breast cancer progression, other biomarkers have been evaluated as prognostic factors in MBC patients [17]. In a study from our team in 250 MBC patients, high serum levels of Neuron Specific Enolase (NSE) and Matrix Metalloproteinase 9 (MMP-9) were associated with poor prognosis in univariate but not in multivariate analysis [17].

The Tau protein, a microtubule-associated protein, is involved in the microtubule stabilization and polymerization. It is mainly localized within the central nervous system (CNS) (mostly in neurons and also in glial cells [20, 21]); its expression has been reported in several cell and tissue types including normal breast epithelial cells [22] and cancer cells [23, 24]. Matrone et al. found that Tau expression is increased in metastatic tissue compared with primary breast cancer cells [25]. Tau expression in tumor tissue has been inconsistently associated with response to taxanes, microtubules-stabilizing agents used in the treatment of breast cancer patients [24, 26]. High Tau protein expression was associated with a more favorable prognosis in breast cancer and MBC in several studies [24, 27]. However, the prognostic value of the serum levels of the Tau protein in cancer patients and more specifically in MBC patients has not been investigated. Considering the different levels of Tau expression between localized and metastatic BC tissues, a circulating evaluation could represent a more accurate evaluation of the MBC cells content and allow a minimally invasive evaluation.

Mainly a neuronal microtubule-stabilizing protein, Tau has been associated with several neurological conditions. Indeed, it plays a major role in the pathophysiology of Alzheimer’s disease (one of the most common “tauopathy”), through its accumulation in a hyper-phosphorylated form (P-Tau). It accumulates (in neurons and/or glial cells) in other neurodegenerative disorders and is released (T-Tau form) in the extracellular matrix and cerebrospinal fluid (CSF) after neuronal damage. Elevated T-Tau CSF levels are found in Alzheimer’s disease [28] and in other diseases such as fronto-temporal dementia [29], multiple sclerosis [30], or following stroke [31]. T-Tau serum levels were also studied in stroke [32] and head traumas [33, 34], and seem associated with the extent of the brain damage [35]. T-Tau thus seems to be a biomarker of neuronal and axonal damage. Despite the fact that brain metastases (BM) are responsible for such damage, CSF or serum T-Tau levels have never been evaluated in this setting so far. Of note, a study on pediatric primary brain tumors revealed that T-Tau CSF levels were significantly higher in these patients compared with controls [36]. In another study, CSF T-Tau levels were found significantly higher in pediatric patients with leukemia-related leptomeningeal involvement [37].

The aim of the present study was to evaluate the prognostic value of several serum tumor markers, namely CA15–3, CEA, T-Tau and HER2 ECD in a series of MBC patients. Also, breast cancer being the second most common cause of BM, we assessed the association of T-Tau serum levels with the presence of BM in MBC patients.

## Methods

### Design

We conducted an exploratory, retrospective, monocentric study on MBC patients treated at our institution between 2007 and 2015 using a previously reported clinico-biological database [17, 38].

### Objectives

The primary objective was to evaluate the prognostic value of serum biomarkers (namely, CA 15–3, CEA, Tau and HER2 ECD) in patients with MBC and in the subpopulation of patients with BM. The secondary objective was to analyze the association of serum levels of Tau with the presence of BM.

### Patients

MBC patients were retrospectively identified by reviewing the medical records of MBC patients from our institution database between 2007 and 2015. Inclusion criteria were as follows: patient ≥18 years old; histologically-confirmed MBC; availability of the HR and HER2 statuses of the primary tumor; and availability of a frozen serum sample performed at the metastatic phase, for biomarker determination. Patients with history of other invasive cancer(s) were excluded.

In order to allow the analysis of the secondary objective regarding the association between serum Tau levels and the presence of BM, and the prognostic value of Tau in patients with BM, our MBC population was enriched with patients diagnosed with BM, as previously published [17]. In this subpopulation of patients, the eligibility criteria were: intraparenchymal BM diagnosed on imaging; availability of a frozen serum sample performed at the time of BM diagnosis (+/− 15 days); and absence of leptomeningeal metastases associated with BM. Patient with BM were matched to patients without BM by age (< 50, 50–70 or > 70), tumor biology (four BC biological subgroups: triple-negative [TN], HR+/HER2-, HR+/HER2+ and HR-/HER2+) and number of previous metastatic treatment lines (0, 1 or 2, > 2).

### Clinical, biological and radiological data

Clinical and biological data were collected by reviewing the medical records of the selected patients: demographical, clinical and biological data (histological grade of the primary tumor, HR and HER2 statuses). The tumor was considered HR-positive when more than 10% of cells were labeled in immunohistochemistry (IHC) or when the concentrations of estrogen (ER) and progesterone receptors (PR) using the radio ligand binding method were above 10 ng/mL and 50 ng/mL, respectively. The tumor was considered HER2-positive if the primary tumor was scored 3+ by IHC or if the HER2 gene was amplified by fluorescence or chromogenic in situ hybridization (FISH/CISH) for IHC 2+ cases. For cases with HR and/or HER2 status changes over time, the biology used was that of the most recent sample. For cases of synchronous or asynchronous bilateral cancer with discrepant HR and/or HER statuses, the most unfavorable biology was used: higher histological grade, HR-negative, HER2-negative (trastuzumab era). The brain imaging with contrast enhancement performed at the time of BM diagnosis (magnetic resonance imaging, MRI or, if not available, CT-scan) was collected for patients with BM in DICOM (*Digital Imaging and Communication in Medicine*) format. The number of BM was determined by a retrospective review of the imaging by AD. BM volume was calculated on post-contrast sequences (T1 sequences for MRI) using the Osirix® software v7.0.2 (Pixmeo, Bernex, Switzerland). For multiples metastases, the volume was calculated as the sum of the volumes of each BM.

### Analysis of serum biomarkers

The selected serum samples were extracted from the Biological Resources Center of our institution (Biobank number BB-0033-00059) (samples processed within one hour and stored at − 80 °C in serum aliquots). The biologists performing the analyses were blinded to the study endpoints. HER2 ECD serum levels were measured by ELISA using commercially available ELISA assays (Nuclea Diagnostic Laboratories kit, LLC) according to the manufacturer’s instructions. The analysis of T-Tau serum levels was performed with a novel immunoassay (digital ELISA) using Single Molecule Array (Simoa) technology (Tau Simoa 2.0 Assay Kit, Quanterix, Lexington, MA, USA) as previously described [39]. Based on singulation of enzyme labeled immune-complex on paramagnetic beads, this assay is associated with a limit of detection of 0.02 pg/mL. Therefore, this method is claimed to be approximately 1000-fold more sensitive than the conventional immunoassay. All samples were analyzed diluted 4-fold with the diluent provided in the kit (phosphate buffer with bovine serum and heterophilic blocker solution) to minimize the matrix effects. The Tau Simoa performances were evaluated and validated within the laboratory by measurement of serum samples (*n* = 72) in duplicates, intra- and inter-assay variations over 10 runs using the low and high quality control (QC) samples provided in the kit. An internal QC of pooled serum sample was included in each experiment (10 runs). As MMP-9 serum level was associated with the presence of BM in a previous study published by our team [38], it was measured by ELISA using commercially-available ELISA assays (Human MMP-9 Quantikine Kit, R&D Systems, Minneapolis, MN, USA). For MMP-9, a duplicate analysis was performed, but no duplicate analyses were deemed necessary for the other biomarkers. Other biological parameters, including CA 15–3 (ELSA-CA15–3 Cisbio assays, Gif sur Yvette, France) and CEA (Elecsys CEA test, Roche Diagnostics, Meylan, France), had previously been analyzed for clinical purposes and were collected. As we evaluated the prognostic value of biomarkers, the manuscript adheres to the REMARK guidelines [40].

### Statistical analysis

Categorical variables were reported: number of missing data, number and percentage for each variable modality. For continuous variables, number of missing data, mean, standard deviation, median and range values were computed.

OS delay was measured from the date of the serum sample to the date of death from any cause. Patients alive without events were censored at the closing date of the study analysis (March 30th, 2015). OS was estimated according to the Kaplan-Meier method, and presented as medians with their 95% confidence intervals (95% CIs), and survival rates in percentages, with 95% CIs [41]. The median duration of follow-up was estimated using a reverse Kaplan-Meier method and presented with its 95% CI. The following cut-offs were used to investigate the prognostic value of the biomarkers: 15 ng/mL for serum HER2 ECD [18], 30 U/mL for CA15–3 and 10 ng/mL for CEA. As there is no validated cut-off for T-Tau in MBC patients, different cut-offs (quartiles, mean) were tested to investigate its prognostic value in the whole population and in patients with BM. To investigate prognostic factors, a multivariate analysis was performed using the Cox’s proportional hazards regression model with a stepwise procedure. Hazard ratios with their 95% CIs were calculated to display risk changes.

Cut-offs were set using the Youden index method associated with receiver-operating characteristics (ROC) curves to evaluate the accuracy of biomarkers to identify patients with BM [42].

To evaluate the correlation between Tau serum levels and BM volumes, the median values of serum Tau were calculated for tumor volumes < 10 cm^3^, ≥10 cm^3^ and non-measurable disease (cases with a high number of small BM) and compared with the Kruskal-Wallis test.

All *p*-values reported were two-sided, and the significance level was set at 5% (*p* < 0.05). Statistical analysis was performed using the STATA 13.1 software (Stata Corporation, College Station, TX).

### Ethical considerations

This study was reviewed and approved by the local Institutional Review Board. Considering the retrospective, non-interventional nature of this study, no additional consent was deemed necessary.

## Results

### Patients’ characteristics

A total of 244 women with MBC were included in the study. The clinical and biological characteristics of patients at baseline are presented in Additional file 1: Table S1. Median age at the time of the serum sample was 58.3 years (range 26.4–87.2). The most represented histological subtype was ductal carcinoma in 83.8% of cases. Tumor biology was distributed as follows: HER2+/HR+ in 25.0%, HER2+/HR- in 23.8%, HER2-/HR+ in 25.4% and triple negative in 25.8% of cases. 29.0% of patients presented with synchronous metastases at first diagnosis of breast cancer (M1). The metastatic-free interval (MFI) was over 24 months for 54.1% of patients. At the time of the serum sample, the median number of previous CT lines was 1 (range 0–9). Among 119 patients with a HER2-positive tumor, 83.2% of patients had received a previous anti-HER2 treatment. A majority of patients had at least four metastatic sites (58.6%). Only 17 patients (7.0%) had only bone and/or subcutaneous metastases. Most patients showed a good performance status (ECOG status ≤2 in 90.7%). Albumin serum level was available for 153 patients and was low in 23 cases (15.0%). Serum LDH level was high in 44.8% of the 87 cases with a reported value.

The characteristics of the subpopulation of patients diagnosed with BM (*n* = 86) are described in Additional file 1: Table S1 and S2. Brain was the first metastatic location in 15 patients (17.4%). The number of BM was one BM in 20.9%, 2–3 in 18.6% and ≥ 4 in 60.5% of patients. The median BM volume (available for 65 cases) was 7.1 cm^3^ (range 0.1–55.3), 0-10 cm^3^ in 53.8% of the cases, >10cm^3^ in 33.8% and non-measurable in 12.3% (eight cases with a high number of small BM). BM location was strictly supratentorial in 20.9%, strictly infratentorial in 15.1% and both supra- and infratentorial in 64.0%. Patients were asymptomatic at BM diagnosis in 34.9% of cases. 45.3% of symptomatic patients presented with intracranial hypertension symptoms, 44.2% with a sensory-motor and/or language deficit, 8.1% with epilepsy and 4.7% with disturbed vigilance. The distribution of BM patients according to the modified breast *Graded Prognostic Assessment* (modified breast GPA) prognosis score [43, 44] was as follows (*n* = 78): 3.5–4 in 9.0% of cases, 2.5–3 in 38.5%, 1.5–2 in 29.5% and 0–1 in 23.1%. A localized treatment (surgery or radiosurgery) was performed in 18.6% of cases: surgery in 8.1% (n = 7, with adjuvant radiotherapy except in one patient) and radiosurgery in 10.5% (*n* = 9). Whole-brain radiotherapy was the most frequent treatment and was performed in 47.7% of the cases. 23.2% of the cases received systemic treatment only, and 10.5% only best supportive care.

### Serum CA 15–3, CEA, tau, HER2 ECD and MMP-9

The median time interval between the first MBC diagnosis and blood collection was 13.9 months for the whole population (14.1 months in the BM group compared with 13.8 months in patients without BM, *p* = 0.78).

Regarding the Simoa approach, the Tau assay evaluation data showed a coefficient of variability (CV) lower than 13% for the Tau serum replicates. Intra and inter assay variations of low and high QC exhibited CV of 3.5 and 13% respectively. The use of an internal QC of pooled serum sample showed intra and inter assay variation with CV of 10.2 and 14% respectively.

The median serum biomarker levels were: 37.5 U/mL (range 8.0–1988.0) for CA 15–3, 4.0 ng/mL (range 1.0–5122.0) for CEA, 1.24 pg/mL (range 0.0–755.5) for T-Tau, 14.0 ng/mL (range 2.8–280.0) for HER2 ECD, and 333.80 ng/mL (range 38.7–2051.0) for MMP-9.

The ROC curves for serum Tau, HER2 ECD and MMP-9 and were calculated to evaluate the ability of each biomarker to identify patients with BM (data not shown). Using the Youden index, the thresholds were calculated as follows: 3.17 pg/mL for Tau, 12.70 ng/mL for HER2 ECD and 245.78 ng/mL for MMP-9.

The proportion of patients with high serum levels is presented for each biomarker in Additional file 1: Table S1. Serum CA 15–3 (cut-off 30 U/mL) and CEA (cut-off 10 ng/mL) were elevated in 60.2 and 33.6% of the 226 patients with known values. Serum Tau was elevated in 23.4% of patients, serum HER2 ECD in 57.4% and serum MMP-9 in 69.7% of patients, respectively (with the cut-offs defined using the Youden index method).

**Fig. 1:**
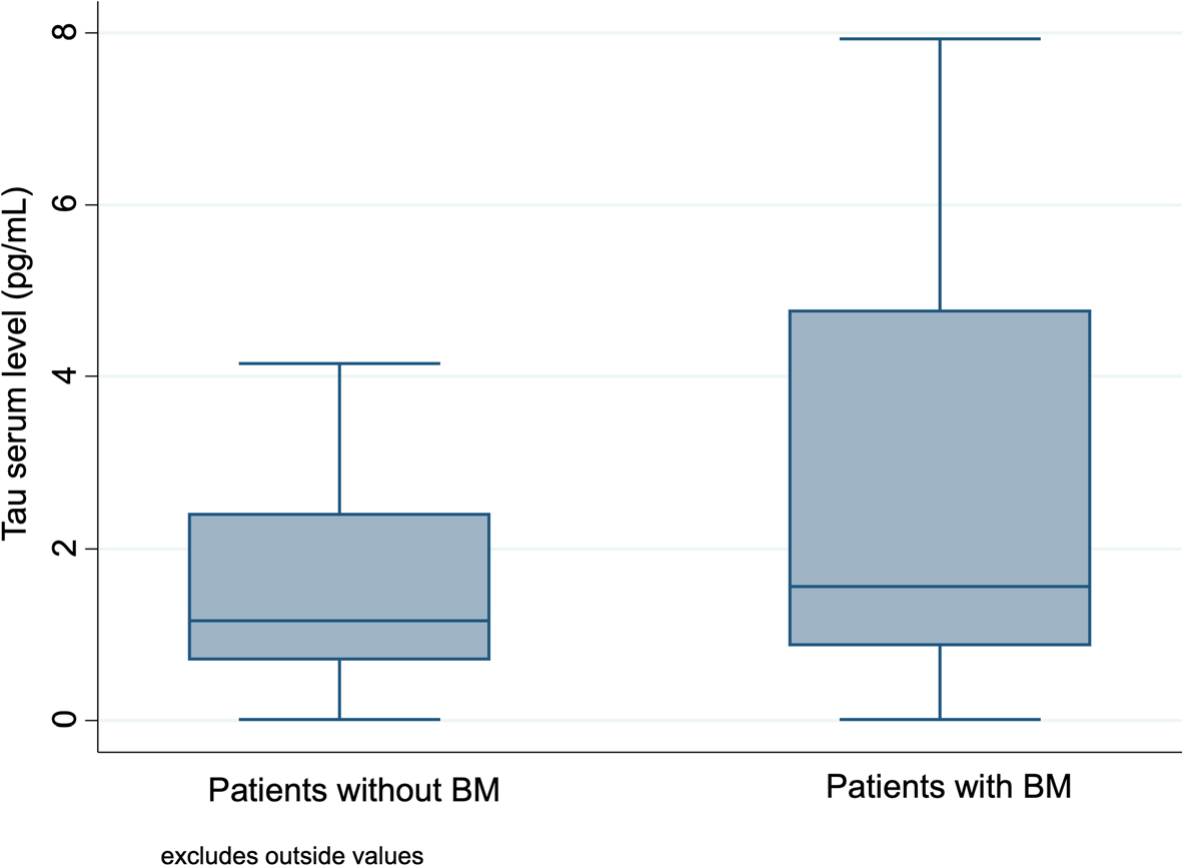
Box-plot of the distribution of Tau serum levels in patients with and without BM

Table 1 summarizes the median and mean values of the studied biomarkers in patients with BM and patients without BM. In patients with BM, the median delay between BM diagnosis and serum sample was 0 days (range − 14 to + 15 days). The distribution of Tau serum levels in patients with and without BM is presented in Fig. 1. Median serum Tau levels were higher in patients with BM (1.56 pg/mL compared with 1.16 pg/mL in patients without BM) but this difference did not reach significance (*p* = 0.23). In univariate analysis, patients with BM had an increased risk of serum Tau > 3.17 pg/mL (OR = 2.2, 95%CI 0.9–5.2, *p* = 0.049). They also had an increased risk of serum HER2 ECD > 12.70 ng/mL (OR = 4.2, 95%CI 2.0–9.3, *p* < 0.001) and MMP-9 > 245.78 ng/mL (OR = 3.4, 95%CI 1.5–8.3, *p* = 0.001). In multivariate analysis, high values of Tau (OR = 3.98, *p* = 0.034) accurately identified patients with BM in our cohort of all MBC patients, as well as high values of HER2 ECD (OR = 7.26, *p* = 0.001), MMP-9 (OR = 4.69, *p* < 0.001) and CEA (OR = 2.71, *p* = 0.030), and low values of CA 15–3 (OR = 3.05, *p* = 0.031).

**Table 1:** Predictive values for the presence of BM of serum CEA, CA 15–3, T-Tau, HER2 ECD and MMP-9 (A) in univariate and (B) multivariate analyses

(A)				
	Total (*n* = 244)	BM (*n* = 86)	No BM (*n* = 158)	*P*-value
CEA (ng/mL) Median (range) Mean (SD)	(*n* = 226) 4.0 (1.0–5122.0) 62.02 (358.1)	(*n* = 81) 9.0 (1.0–5122.0) 122.72 (582.7)	(*n* = 145) 3.0 (1.0–768.0) 28.12 (92.1)	0.025
CA 15–3 (U/mL) Median (range) Mean (SD)	(*n* = 226) 37.5 (8.0–1988.0) 177.27 (337.6)	(*n* = 81) 48.0 (11.0–1549.0) 210.92 (324.8)	(*n* = 145) 34.0 (8.0–1988.0) 158.48 (344.3)	0.157
T-Tau (pg/mL) Median (range) Mean (SD)	(*n* = 244) 1.24 (0.0–755.5) 18.70 (92.2)	(*n* = 86) 1.56 (0.0–751.1) 30.60 (120.4)	(*n* = 158) 1.16 (0.0–755.5) 12.22 (72.1)	0.232
HER2-ECD (ng/mL) Median (range) Mean (SD)	(*n* = 244) 14.00 (2.8–280) 36.87 (58.10)	(*n* = 86) 21.30 (2.8–280) 49.01 (68.20)	(*n* = 158) 12.15 (5.7–280) 30.26 (50.90)	0.077
MMP-9 (ng/mL) Median (range) Mean (SD)	(*n* = 244) 333.80 (38.7–2051) 415.43 (300.70)	(*n* = 86) 416.08 (38.7–1575.8) 463.83 (292.2)	(*n* = 158) 317.00 (39–2051) 389.08 (302.8)	0.066
(B)				
	Hazard-ratio	95%CI	*P*-value	
CEA	2.71	1.10–6.68	0.030	
CA 15–3	0.33	0.14–0.78	0.012	
T-Tau	3.98	1.11–14.30	0.034	
HER2 ECD	7.26	2.32–22.71	0.001	
MMP-9	4.69	2.05–10.73	< 0.001	

Abbreviations: BM: brain metastases; CEA: Carcinoembryonic Antigen; CA 15–3: Cancer Antigen 15–3; HER2-ECD: HER2-extra-cellular domain; MMP-9: Matrix Metalloproteinase 9

The median values of serum Tau were 1.85 pg/mL (range 0.01–427.3) for tumor volumes < 10 cm^3^, 1.24 pg/mL (range 0.22–751.1) for tumor volumes ≥10 cm^3^ and 1.57 pg/mL (range 0.06–17.6) for non-measurable disease (*p* = 0.82).

### Prognostic factors

**Fig. 2:**
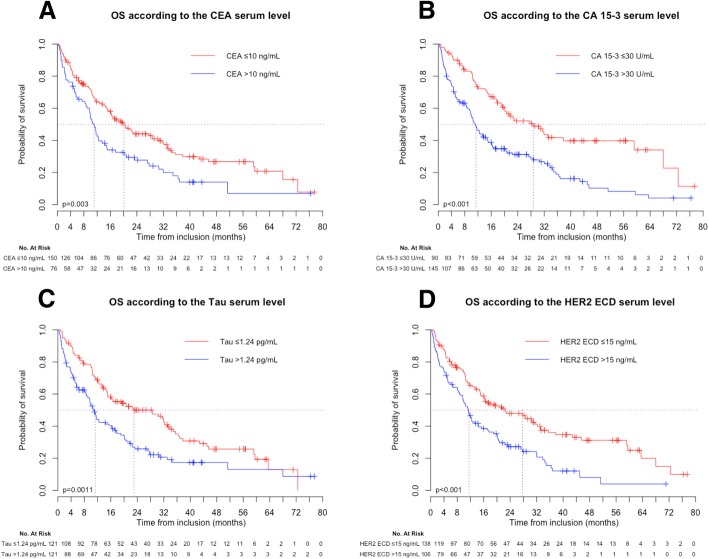
Overall survival (OS) in the whole MBC population (*n* = 244) according to (**a**) the CEA serum level, (**b**) the CA 15–3 serum level, (**c**) the T-Tau serum level and (**d**) the HER2 ECD serum level. * NC: not calculable

At the time of the analysis, 69.7% of patients had died, mostly because of MBC progression (88.2%). This proportion was significantly higher when considering patients diagnosed with BM (90.7%, *p* < 0.0001). With a median follow-up of 40.8 months (95% CI 31.1–43.6), median OS was 15.5 months (95% CI 12.4–20.2). In patients with BM (median follow-up 40.9 months, 95% CI 28.2-NC), median OS was 9.7 months (95% CI 5.6–10.8), compared with 30.4 months (95% CI 16.8–34.5) in patients without BM (*p* < 0.0001). The 1-year and 2-year survival rates in the entire population (*n* = 244) were 57.1% (95% CI 50.5–63.1) and 38.2% (95% CI 31.8–44.6), respectively. Table 2 and Additional file 1: Table S3 summarize the variables significantly associated with prognosis in the univariate analysis in the entire population. Among the tested biomarkers, high levels of CEA (*p* = 0.003), CA 15–3 (*p* < 0.001), Tau (*p* = 0.0011, cut-off corresponding to the median, 1.24 pg/mL) and HER2 ECD (*p* < 0.001) were also poor prognostic determinants in univariate analysis (Fig. 2). The prognostic significance of the serum level of Tau remained true whatever the cut-off used: first quartile (0.74 pg/mL, *p* = 0.007), third quartile (3.17 pg/mL, *p* < 0.001) or mean (18.70 pg/mL, *p* = 0.035). A multivariate analysis was performed (*n* = 210), excluding albumin due to a high number of missing data. The patient’s age at the time of the serum sample, ECOG performance status, presence of BM or subcutaneous metastases, number of previous metastatic CT lines, tumor biological subtype, high HER2 ECD, high CA 15–3 and high Tau serum levels were independently associated with poor prognosis (Table 3). These results, in particular those regarding the prognostic value of the Tau serum levels (*p* = 0.016), were unchanged when the tumor biology was categorized in three groups (HER2+, HER2-/HR+, and triple-negative tumors) instead of four.

**Table 2:** Univariate analysis of OS (A) in the whole MBC population (*n* = 244) and (B) in the BM population (*n* = 86): main results

	Whole MBC population (*n* = 244)		BM population (*n* = 86)	
Parameter	Median OS in months (95 CI%)	*P*-value	Median OS in months (95 CI%)	*P*-value
Initial characteristics				
* Tumor biology group* HER2+ HR+ HER2+ HR- HER2- HR+ Triple negative	28.6 (16.5 – NC) 31.6 (20.2–34.5) 12.4 (10.4–22.5) 6.3 (4.5–10.4)	< 0.001	14.4 (8.1–20.1) 8.7 (2.6–20.2) 9.7 (2.6–16.2) 4.6 (2.0–8.5)	0.0598
* PR status* Negative Positive	12.5 (10.6–17.8) 21.7 (16.2–35.6)	0.044	8.1 (4.6–10.4) 16.2 (9.7–21.7)	0.119
* HER2 status* Negative Positive	10.4 (8.4–12.4) 28.7 (20.1–33.9)	< 0.001	6.4 (4.2–10.0) 12.1 (8.1–19.1)	0.037
* Histological grade (SBR)* 1 or 2 3	20.9 (15.1–31.6) 11.1 (9.5–15.0)	0.012	10.4 (6.4–20.6) 8.1 (4.2–10.4)	0.117
* Metastatic status at BC diagnosis* M0 M1	13.6 (10.5–16.5) 27.2 (13.7–32.1)	0.016	8.5 (4.7–10.0) 11.6 (4.2–20.9)	0.056
* Adjuvant or néoadjuvant CT* No Yes	20.7 (16.8–31.6) 12.5 (10.3–15.5)	0.009	11.6 (8.4–20.3) 6.8 (3.3–10.3)	0.007
Characteristics at the time of the serum sample				
* ECOG status* Score 0 Score 1 Score 2 Score 3	34.5 (20.9–63.6) 16.5 (12.1–22.5) 6.8 (3.8–16.8) 2.0 (1.4–2.6)	< 0.001	14.4 (10.0 – NC) 12.1 (8.4–19.1) 8.5 (1.5–17.6) 2.0 (1.2–4.6)	< 0.001
* Number of lines of CT* 0 line 1 or 2 line(s) > 2 lines	17.8 (15.0–27.8) 20.9 (16.3–32.0) 6.4 (4.6–10.4)	< 0.001	8.5 (4.7–17.6) 12.4 (8.4–20.3) 4.6 (2.2–9.7)	0.007
* Number of metastatic sites* 1–3 ≥4	30.7 (19.1–59.2) 11.7 (9.9–15.0)	< 0.001	10.3 (5.6–19.1) 9.7 (4.7–12.1)	0.309
* Location of metastatic sites* Bone and/or subcutaneous only Visceral	63.6 (16.5 – NC) 14.4 (11.4–19.1)	0.0016	NC 9.7 (5.6–10.8)	0.843
* Brain metastases* Absent Present	30.4 (16.8–34.5) 9.7 (5.6–10.8)	< 0.001	- -	–
* Liver metastases* Absent Present	28.7 (14.9–36.8) 12.4 (10.6–17.2)	< 0.001	6.8 (3.8–10.4) 9.8 (4.9–13.6)	0.377
* Subcutaneous metastases* Absent Present	19.2 (13.7–22.6) 10.4 (4.6–15.2)	0.0034	10.0 (8.1–13.6) 4.7 (1.4–9.8)	0.011
* Metastases of other sites* Absent Present	20.9 (16.2–30.7) 10.6 (6.4–12.5)	< 0.001	10.0 (8.1–15.5) 5.3 (2.8–11.6)	0.876
* Serum albumin level* Normal Low	14.4 (11.1–19.4) 1.6 (1.1–2.4)	< 0.001	10.0 (6.8–12.4) 1.4 (0.7–2.6)	< 0.001
* Serum CEA* Normal Elevated	20.1 (15.5–28.6) 11.1 (8.7–13.6)	0.003	10.0 (4.7–15.5) 9.7 (3.3–12.1)	0.483
* Serum CA 15–3* Normal Elevated	28.7 (19.4–59.2) 11.4 (9.7–14.4)	< 0.001	10.4 (6.8–20.1) 8.5 (4.0–10.4)	0.096
* Serum HER2 ECD (cut-off 15 ng/mL)* Normal Elevated	21.7 (15.2–31.6 11.4 (8.9–14.4)	< 0.001	10.3 (4.7–21.7) 8.7 (4.6–12.1)	0.085
Serum Tau (cut-off 0.74 pg/mL*) Normal Elevated	33.2 (19.2–42.8) 12.4 (10.6–16.3)	0.007	12.1 (6.8–28.6) 8.5 (4.6–10.4)	0.060
Serum Tau (cut-off 1.24 pg/mL**) Normal Elevated	23.0 (15.0–33.2) 11.3 (9.5–16.3)	0.0011	10.3 (4.6–12.4) 8.7 (4.9–12.4)	0.942
Serum Tau (cut-off 3.17 pg/mL***) Normal Elevated	20.6 (16.2–30.4) 9.5 (4.9–10.8)	< 0.001	10.3 (5.3–13.6) 8.7 (2.6–10.8)	0.389

Abbreviations: PR: progesterone-receptors; SBR: Scarf, Bloom and Richardson; BC: breast cancer; CT: chemotherapy; CEA: Carcinoembryonic Antigen; CA 15–3: Cancer Antigen 15–3; HER2-ECD: HER2-extra-cellular domain; NC: not calculated; NSE: Neuron Specific Enolase; MMP-9: Matrix Metalloproteinase 9

*cut-off corresponding to the first quartile

**cut-off corresponding to the median

***cut-off corresponding to the third quartile

**Table 3:** Multivariate Cox regression analyses (Stepwise procedure) (A) in the whole MBC population (*n* = 244) and (B) in the BM population (*n* = 86)

	Whole MBC population (*n* = 244)			BM population (*n* = 86)		
Parameter	Hazard-ratio	95% CI	*P*-value	Hazard-ratio	95% CI	*P*-value
Performance status						
ECOG 0	1			1		
ECOG 1	1.90	1.17–3.07	0.009	1.58	0.65–3.85	0.316
ECOG 2	2.66	1.44–4.90	0.002	1.63	0.54–4.96	0.385
ECOG 3	11.82	5.98–23.36	< 0.001	11.16	3.50–35.63	< 0.001
Tumor biology						
HER2+ / HR+	1			1		
HER2+ / HR-	0.97	0.56–1.66	0.910	1.41	0.67–2.96	0.360
HER2- / HR+	1.72	1.09–2.71	0.020	2.30	1.16–4.56	0.017
Triple negative	6.50	3.63–11.63	< 0.001	2.54	1.00–6.47	0.050
Patient’s age						
< 50	1			1		
50–70	1.67	1.08–2.59	0.022	2.00	1.15–3.45	0.013
≥71	1.85	0.94–3.64	0.077	2.06	0.90–4.72	0.088
Number of previous metastatic CT lines						
0	1			–		
1 or 2	1.53	0.95–2.47	0.080			
> 2	2.50	1.52–4.12	< 0.001			
Adjuvant or neoadjuvant CT	–			1.44	0.88–2.38	0.148
Brain metastases	2.03	1.39–2.97	< 0.001	–		
Neurological deficit associated with BM	–			2.75	1.50–5.03	< 0.001
Subcutaneous metastases	1.85	1.26–2.72	0.002	2.87	1.64–5.04	< 0.001
Elevated HER2 ECD (cut-off 15 ng/mL)	1.88	1.21–2.91	0.005	2.24	1.13–4.43	0.007
Elevated Tau (cut-offs 1.24 and 0.74 pg/mL, respectively)	1.58	1.09–2.31	0.017	2.43	1.16–5.09	0.018

Abbreviations: ECOG: Eastern Cooperative Oncology Group; CT: chemotherapy: CA 15–3: Cancer Antigen 15–3; HER2-ECD: HER2-extra-cellular domain

**Fig. 3:**
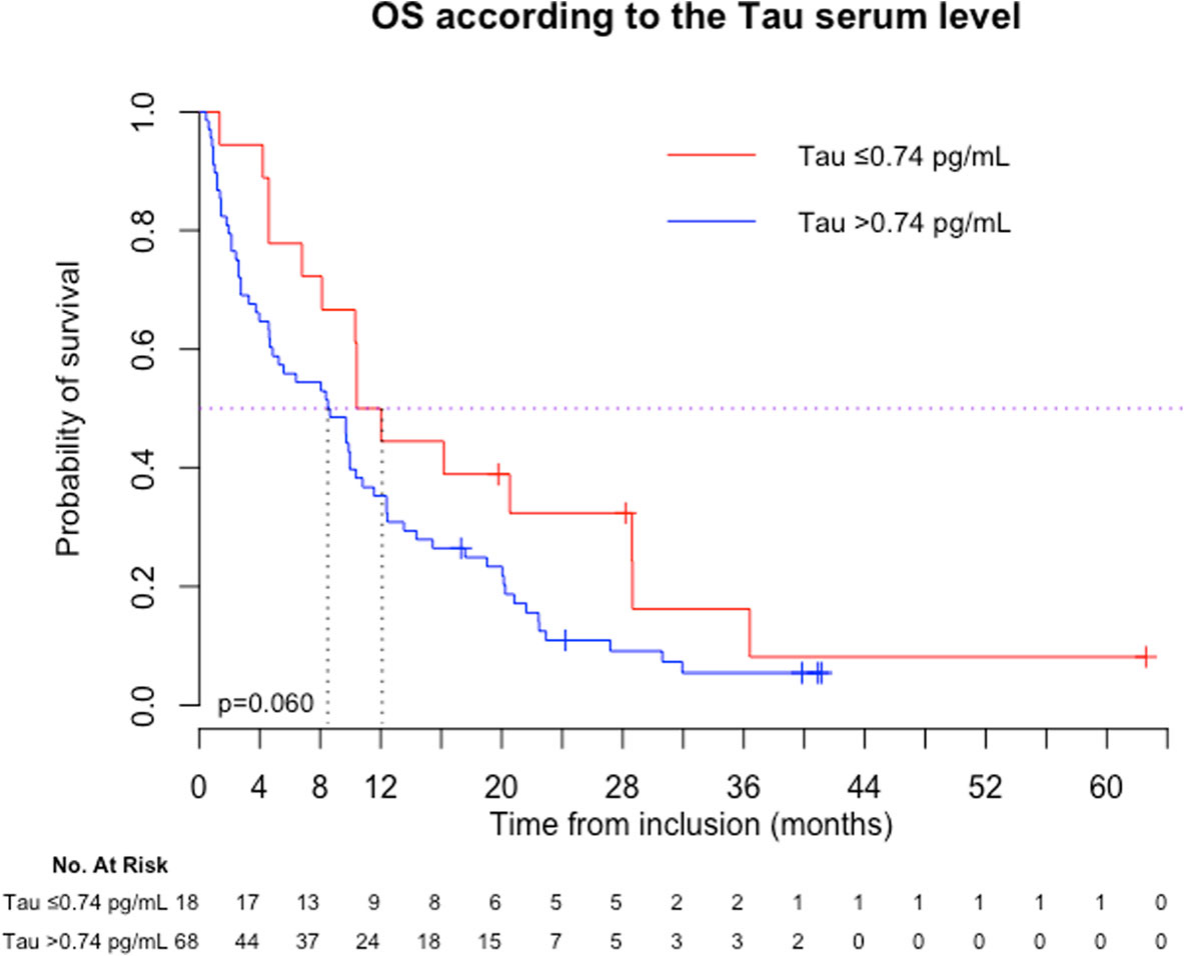
Overall survival (OS) in the BM population (*n* = 86) according to the T-Tau serum level

The variables significantly associated with poor prognosis in univariate analysis in patients with BM (*n* = 86) are presented in Table 2. Among the tested biomarkers, only high serum Tau level showed a trend (*p* = 0.060, using the first quartile cut-off) towards an association with prognostic in univariate analysis (Fig. 3). A multivariate analysis was performed (*n* = 78), excluding serum proteins and albumin due to a high number of missing data. The patient’s age at the time of the serum sample, ECOG performance status, presence of subcutaneous metastases, tumor biological subtype and the presence of a neurological deficit associated with BM were independently associated with poor prognosis. Among the tested biomarkers, high serum levels of HER2 ECD and Tau were independently associated with a poor outcome (Table 3).

## Discussion

### Association of tau serum level and brain metastases

In this series of 244 MBC patients, we found an association between Tau serum level and the presence of BM. In univariate analysis, patients diagnosed with BM had an increased risk of having a higher Tau serum level. This was confirmed in multivariate analysis: Tau serum level was independently associated with the presence of BM (OR = 3.98, *p* = 0.034). However, the results of our multivariate model must be considered with caution as, quite surprisingly, the relation of CA 15–3 serum level with the presence of BM was inversed, patients with BM having an increased risk of having a lower CA 15–3.

To our knowledge, this is the first report of such a correlation between Tau serum level and the presence of a brain tumor. So far, only one report has been published of an association between the levels of Tau in CSF and brain tumors in pediatric patients [36, 45], while several reports of correlations with Tau have been published for other non-neurodegenerative disorders, including traumatic brain injury (TBI) [34, 33] and stroke [46]. In TBI, the serum level of Tau was associated with the presence of macroscopic lesions detectable on brain imaging, and with the patients’ clinical outcome [33]. Tau was also associated with the clinical severity and the infarct volume in stroke patients [46].

In our study, there was no correlation between the serum levels of Tau and the BM volume measured on post-contrast imaging, in contrast with data reported in TBI or stroke. Several hypotheses can be discussed to explain this finding. First, we chose to measure BM volume on post-contrast imaging (measurement of the enhancement), therefore we did not take into account the surrounding edema that could also cause neuronal damage. Moreover, measures were performed on images from various devices (CT-scan, MRI), which could also be a source of bias. Second, the correlation analysis was limited by the relatively reduced number of patients with measurable BM. Of 86 patients with BM, imaging was available in DICOM format for 65 patients only. Among them, 8 presented with a high number of small BM, not accessible to BM volume calculation. Finally, it is possible that there is really no correlation between Tau and BM volume.

Our data suggests an association between the serum levels of Tau and the presence of BM in MBC patients. However, due to the limitations of our multivariate model and to the absence of correlation with BM volume, this result needs to be confirmed.

### Prognostic value of tau serums level

To our knowledge, this study is the first report of an association between the serum levels of Tau and survival in MBC patients. However, a few studies have evaluated the prognostic value of Tau expression in tumor tissue in breast cancer [24, 26, 27]. Indeed, Tau expression seems to be higher in MBC compared to that reported in early breast cancer [25]. In 102 patient-matched primary and metastatic samples, Tau expression was found in 26 and 52%, respectively. The prognostic value of Tau expression in tumor tissue has been suggested in several studies [26], including one performed in 54 patients treated with paclitaxel and cisplatin as first-line chemotherapy for locally-advanced or metastatic breast cancer [27]. High expression of Tau was associated with longer OS in the whole population of breast cancer patients as well as in patients with MBC. This prognostic advantage could be linked with an increased microtubule stability, leading to less aggressive tumors. Of note, a predictive value of Tau expression in tumor tissue for the tumor response to taxanes has been inconsistently reported in metastatic or early breast cancer [26].

For the first time, the prognostic value of Tau serum level was also found specifically in the subpopulation of patients diagnosed with BM. So far, only few studies have reported a prognostic impact of Tau measured in CSF, but not serum, in brain tumors. Indeed, CSF Tau was elevated in pediatric patients with CNS malignancies compared with pediatric patients without CNS [36]. Other studies have evaluated the prognostic impact of Tau measured in serum and non-tumoral neurological disorders such as TBI [34] or stroke [46].

The mechanisms underlying the prognostic impact of Tau serum levels in MBC patients need to be clarified. First, it could be hypothesized that it results from the enrichment of our population with patients diagnosed with BM. Indeed, Tau is a known biomarker of axonal damage. As discussed before, our data suggests an association between the presence of BM and elevated serum values of Tau, and Tau seems predictive of OS in MBC patients with BM. Therefore, our MBC population being enriched with BM patients, the prognostic value of Tau could be a result of its association with OS in BM patients. To test this hypothesis, we analyzed survival in the subpopulation of patients without BM (*n* = 158) to see if serum Tau remained associated with OS. High serum level of Tau was indeed associated with poor OS in univariate and multivariate analysis (data not shown, median OS 14.4 vs. 34.9 months, *p* = 0.004, using the median value of Tau as a cut-off). Therefore, the prognostic value of serum Tau does not seem to be limited to patients with BM. Additionally, the prognostic impact of Tau in the whole MBC population remains significant in multivariate analysis, independently of the presence of BM. One could argue that BM might have been undiagnosed in a proportion of our patients, since no systematic brain imaging is performed in the absence of symptoms at our institution, and BM incidence has been reported to be higher in pathological series (18% of patients [47]) than in clinical series (5.1% [48]). However, in asymptomatic patients with BM, one would expect small BM volumes, responsible for only slight neuronal damage.

Other mechanisms independent of brain damage could also be involved. In further exploratory analysis, we found a significant association between serum HER2 ECD and serum Tau (Pearson correlation coefficient 0.182, *p* = 0.004). One could thus argue that HER2 ECD mediates the prognostic impact of Tau. Indeed, its poor prognostic value in MBC patients was demonstrated [17]. Moreover, the MAPT gene (encoding for the Tau protein) and the ERBB2 gene are closely located on the 17q21 chromosome, and could be co-amplified. However, we found no correlation between the presence of HER2 amplification and the serum value of Tau in our patients.

Of note, the inverse prognostic value of serum Tau compared with its tissue expression could be linked to the fact that these two analyses do not measure the same thing. While the tissue expression of Tau seems to favor the integrity of microtubules (and thus improve the prognosis), the release of Tau in serum reflects tissue damage (not only in the brain but also in other tissues such as muscles, vessels, kidney…).

### Limitations

Our study is based on a well-characterized series of MBC patients and brings new data on the prognostic value of the Tau protein serum levels. Nevertheless, it has some limitations. Due to its retrospective nature, we could not avoid missing data, in particular for classical biological parameters such as LDH or albumin. Moreover, because we chose to enrich our population with patients diagnosed with BM as to allow the analysis of the association between serum Tau levels and the presence of BM and the prognostic value of Tau in patients with BM, our population may not be entirely representative of a routine MBC population. In particular, there is in our series an over-representation of patients with a HER2-positive tumor. Regarding the Simoa approach, we reduced the variability by measuring the samples in batches and using a single reagent lot. The levels of Tau detected were above the limit of quantification, which ensure the quality of the result. The Tau Simoa assay showed low assay variations for the serum samples replicates with CVs lower than 10% in accordance with the laboratory’s criteria of immunoassay evaluation (CV < 20%). It is possible however that the delay of storage of the sample impacts the measure, but this possible bias is present for the all population and cannot account for the differences observed. Finally, we acknowledge that our results need to be confirmed in an independent cohort. Performing a cross-validation procedure to validate our data was unfortunately not possible due to the relatively limited number of patients in the BM group.

## Conclusions

In conclusion, the Tau protein is a new biomarker of interest in MBC. Its serum level seems to represent an independent prognostic factor in these patients (both in patients with and without BM). Tau serum level also seems to be associated with the presence of BM in this population. A validation of these results in an independent set of MBC samples is necessary to confirm these findings, in particular because of the inversed behavior of CA 15–3 serums levels with the presence of BM. Further confirmatory studies are mandatory.

## Additional file

Additional file 1: **Table S1.** Patients’ clinical and biological characteristics. **Table S2**. Characteristics of brain metastases. **Table S3.** Univariate analysis of OS (A) in the whole MBC population (*n* = 244) and (B) in the BM population (*n* = 86): additional results. (DOCX 75 kb)

## Abbreviations

BM: brain metastases
CA 15–3: cancer antigen 15–3
CEA: carcinoembryonic antigen
CI: confidence interval
CISH: chromogenic in situ hybridization
CNS: central nervous system
CSF: cerebrospinal fluid
CT: chemotherapy
CV: coefficient of variation
DICOM: Digital Imaging and Communication in Medicine
ER: estrogen receptors
FISH: fluorescence in situ hybridization
HER 2 ECD: HER2 extra-cellular domain
HER2: human epidermal growth factor 2
HR: hormone-receptors
IHC: immunohistochemistry
LDH: lactate dehydrogenase
MBC: metastatic breast cancer
MMP-9: matrix metalloproteinase 9
NSE: neuron specific enolase
OR: odd-ratio
OS: overall survival
PR: progesterone receptors
QC: quality control
ROC: receiver-operating characteristics
TBI: traumatic brain injury.

## References

[1] Chia SK, Speers CH, D'yachkova Y, Kang A, Malfair-Taylor S, Barnett J (2007). The impact of new chemotherapeutic and hormone agents on survival in a population-based cohort of women with metastatic breast cancer. Cancer.

[2] Mosconi P, Colozza M, De Laurentiis M, De Placido S, Maltoni M (2001). Survival, quality of life and breast cancer. Ann Oncol.

[3] Andreopoulou E, Hortobagyi GN (2008). Prognostic factors in metastatic breast cancer: successes and challenges toward individualized therapy. J Clin Oncol.

[4] Largillier R, Ferrero JM, Doyen J, Barriere J, Namer M, Mari V (2008). Prognostic factors in 1,038 women with metastatic breast cancer. Ann Oncol.

[5] Lobbezoo DJ, van Kampen RJ, Voogd AC, Dercksen MW, van den Berkmortel F, Smilde TJ (2013). Prognosis of metastatic breast cancer subtypes: the hormone receptor/HER2-positive subtype is associated with the most favorable outcome. Breast Cancer Res Treat.

[6] Solomayer EF, Diel IJ, Meyberg GC, Gollan C, Bastert G (2000). Metastatic breast cancer: clinical course, prognosis and therapy related to the first site of metastasis. Breast Cancer Res Treat.

[7] Beslija S, Bonneterre J, Burstein HJ, Cocquyt V, Gnant M, Heinemann V (2009). Third consensus on medical treatment of metastatic breast cancer. Ann Oncol.

[8] Regierer AC, Wolters R, Ufen MP, Weigel A, Novopashenny I, Köhne CH (2014). An internally and externally validated prognostic score for metastatic breast cancer: analysis of 2269 patients. Ann Oncol.

[9] Kennecke H, Yerushalmi R, Woods R, Cheang MC, Voduc D, Speers CH (2010). Metastatic behavior of breast cancer subtypes. J Clin Oncol.

[10] Dawood S, Broglio K, Buzdar AU, Hortobagyi GN, Giordano SH (2010). Prognosis of women with metastatic breast cancer by HER2 status and trastuzumab treatment: an institutional-based review. J Clin Oncol.

[11] Yamamoto N, Watanabe T, Katsumata N, Omuro Y, Ando M, Fukuda H (1998). Construction and validation of a practical prognostic index for patients with metastatic breast cancer. J Clin Oncol.

[12] Wyld L, Gutteridge E, Pinder SE, James JJ, Chan SY, Cheung KL (2003). Prognostic factors for patients with hepatic metastases from breast cancer. Br J Cancer.

[13] Brown JE, Cook RJ, Lipton A, Coleman RE (2012). Serum lactate dehydrogenase is prognostic for survival in patients with bone metastases from breast cancer: a retrospective analysis in bisphosphonate-treated patients. Clin Cancer Res.

[14] Bidard FC, Hajage D, Bachelot T, Delaloge S, Brain E, Campone M (2012). Assessment of circulating tumor cells and serum markers for progression-free survival prediction in metastatic breast cancer: a prospective observational study. Breast Cancer Res.

[15] Lee JS, Park S, Park JM, Cho JH, Kim SI, Park BW (2013). Elevated levels of serum tumor markers CA 15-3 and CEA are prognostic factors for diagnosis of metastatic breast cancers. Breast Cancer Res Treat.

[16] Shao Y, Sun X, He Y, Liu C, Liu H (2015). Elevated levels of serum tumor markers CEA and CA15-3 are prognostic parameters for different molecular subtypes of breast Cancer. PLoS One.

[17] Darlix A, Lamy PJ, Lopez-Crapez E, Braccini AL, Firmin N, Romieu G, Thezenas S, Jacot W (2016). Serum HER2 extra-cellular domain, S100ß and CA 15-3 levels are independent prognostic factors in metastatic breast cancer patients. BMC Cancer.

[18] Tsé C, Gauchez AS, Jacot W, Lamy PJ (2012). HER2 shedding and serum HER2 extracellular domain: biology and clinical utility in breast cancer. Cancer Treat Rev.

[19] Baselga J, Cortés J, Im SA, Clark E, Ross G, Kiermaier A (2014). Biomarker analyses in CLEOPATRA: a phase III, placebo-controlled study of pertuzumab in human epidermal growth factor receptor 2-positive, first-line metastatic breast cancer. J Clin Oncol.

[20] Binder LI, Frankfurter A, Rebhun LI (1985). The distribution of tau in the mammalian central nervous system. J Cell Biol.

[21] Johnson GV, Bailey CD (2002). Tau, where are we now?. J Alzheimers Dis.

[22] Rouzier R, Rajan R, Wagner P, Hess KR, Gold DL, Stec J, Ayers M, Ross JS, Zhang P, Buchholz TA, Kuerer H, Green M, Arun B, Hortobagyi GN, Symmans WF, Pusztai L (2005). Microtubule-associated protein tau: a marker of paclitaxel sensitivity in breast cancer. Proc Natl Acad Sci U S A.

[23] Souter S, Lee G (2009). Microtubule-associated protein tau in human prostate cancer cells: isoforms, phosphorylation, and interactions. J Cell Biochem.

[24] Baquero MT, Lostritto K, Gustavson MD, Bassi KA, Appia F, Camp RL, Molinaro AM, Harris LN, Rimm DL (2011). Evaluation of prognostic and predictive value of microtubule associated protein tau in two independent cohorts. Breast Cancer Res.

[25] Matrone MA, Whipple RA, Thompson K, Cho EH, Vitolo MI, Balzer EM, Yoon JR, Ioffe OB, Tuttle KC, Tan M, Martin SS (2010). Metastatic breast tumors express increased tau, which promotes microtentacle formation and the reattachment of detached breast tumor cells. Oncogene.

[26] Bonneau C, Gurard-Levin ZA, Andre F, Pusztai L, Predictive RR (2015). Prognostic value of the TauProtein in breast Cancer. Anticancer Res.

[27] Shao YY, Kuo KT, Hu FC, Lu YS, Huang CS, Liau JY, Lee WC, Hsu C, Kuo WH, Chang KJ, Lin CH, Cheng AL (2010). Predictive and prognostic values of tau and ERCC1 in advanced breast cancer patients treated with paclitaxel and cisplatin. Jpn J Clin Oncol.

[28] Sunderland T, Linker G, Mirza N (2003). Decreased beta-amyloid1-42 and increased tau levels in cerebrospinal fluid of patients with Alzheimer disease. JAMA.

[29] Riemenschneider M, Wagenpfeil S, Diehl J (2002). Tau and Abeta42 protein in CSF of patients with frontotemporale degeneration. Neurology.

[30] Bartosik-Psujek H, Archelos JJ (2004). Tau protein and 14-3-3 are elevated in the cerebrospinal fluid of patients with multiple sclerosis ans correlate with intrathecal synthsis of IgG. J Neurol.

[31] Hesse C, Rosengren L, Vanmechelen E (2000). Cerebrospinal fluid markers for Alzheimer’s disease evaluated after acute ischemic stroke. J Alzheimers Dis.

[32] Bitsch A, Horn C (2002). Kemmling yet al. Serum tau protein level as a marker of axonal damage in acute ischemic stroke. Eur Neurol.

[33] Shaw GJ, Jauch EC, Zemlan FP (2002). Serum cleaved tau protein levels and clinical outcome in adult patients with closed head injury. Ann Emerg Med.

[34] Shahim P, Blennow K, Zetterberg H (2014). Tau, s-100 calcium-binding protein B, and neuron-specific enolase as biomarkers of concussion-reply. JAMA Neurol.

[35] Ahmad O, Wardlaw J, Whiteley WN (2012). Correlation of levels of neuronal and glial markers with radiological measures of infarct volume in ischaemic stroke: a systematic review. Cerebrovasc Dis.

[36] de Bont JM, Vanderstichele H, Reddingius RE (2008). Increased total-tau levels in cerebrospinal fluid of pediatric hydrocephalus and brain tumor patients. Eur J Paediatr Neurol.

[37] Van Gool SW, Van Kerschaver E, Brock P (2000). Disease- and treatment-related elevation of the neurodegenerative marker tau in children with hematological malignancies. Leukemia.

[38] Darlix A, Lamy PJ, Lopez-Crapez E, Braccini AL, Firmin N, Romieu G, Thézenas S, Jacot W (2016). Serum NSE, MMP-9 and HER2 extracellular domain are associated with brain metastases in metastatic breast cancer patients: predictive biomarkers for brain metastases?. Int J Cancer.

[39] Rissin DM, Kan CW, Campbell TG, Howes SC, Fournier DR, Song L, Piech T, Patel PP, Chang L, Rivnak AJ, Ferrell EP, Randall JD, Provuncher GK, Walt DR, Duffy DC (2010). Single-molecule enzyme-linked immunosorbent assay detects serum proteins at subfemtomolar concentrations. Nat Biotechnol.

[40] McShane LM, Altman DG, Sauerbrei W, Taube SE, Gion M, Clark GM (2006). Statistics subcommittee of NCI-EORTC working group on Cancer diagnostics. REporting recommendations for tumor MARKer prognostic studies (REMARK). Breast Cancer Res Treat.

[41] Kaplan EL, Meier P (1958). Nonparametric estimation from incomplete observations. J Am Stat Assoc.

[42] Youden WJ (1950). Index for rating diagnostic tests. Cancer.

[43] Subbiah IM, Lei X, Weinberg JS, Sulman EP, Chavez-MacGregor M, Tripathy D, Gupta R, Varma A, Chouhan J, Guevarra RP, Valero V, Gilbert MR, Gonzalez-Angulo AM (2015). Validation and development of a modified breast graded prognostic assessment as a tool for survival in patients with breast Cancer and Brain metastases. J Clin Oncol.

[44] Griguolo G, Jacot W, Kantelhardt E, Dieci MV, Bourgier C, Thomssen C, Bailleux C, Miglietta F, Braccini AL, Conte P, Ferrero JM, Guarneri V, Darlix A (2017). External validation of modified breast graded prognostic assessment for breast cancer patients with brain metastases: a multicentric European experience. Breast.

[45] Cengiz P, Zemlan F, Eickhoff JC, Ellenbogen R, Zimmerman JJ (2015). Increased cerebrospinal fluid cleaved tau protein (C-tau) levels suggest axonal damage in pediatric patients with brain tumors. Childs Nerv Syst.

[46] Wunderlich MT, Lins H, Skalej M, Wallesch CW, Goertler M (2006). Neuron-specific enolase and tau protein as neurobiochemical markers of neuronal damage are related to early clinical course and long-term outcome in acute ischemic stroke. Clin Neurol Neurosurg.

[47] Tsukada Y, Fouad A, Pickren JW, Lane WW (1983). Central nervous system metastasis from breast carcinoma. Autopsy study. Cancer.

[48] Davis FG, Dolecek TA, McCarthy BJ, Villano JL (2012). Toward determining the lifetime occurrence of metastatic brain tumors estimated from 2007 United States cancer incidence data. Neuro-Oncology.

