# Supplementary material for: The prognostic value of the Tau protein serum level in metastatic breast cancer patients and its correlation with brain metastases
Source: BMC Cancer. 2019 Jan 30;19:110. doi: 10.1186/s12885-019-5287-z (PMC6354387; doi:10.1186/s12885-019-5287-z)
Supplement: Supplementary file 1 — Table S1. Patients’ clinical and biological characteristics. Table S2. Characteristics of brain metastases. Table S3. Univariate analysis of OS (A) in the whole MBC population (n = 244) and (B) in the BM population (n = 86): additional results. (DOCX 75 kb) [file 12885_2019_5287_MOESM1_ESM.docx]

**Supplementary Table 1**. Patients’ clinical and biological characteristics.

|  | **Whole MBC population (n=244)** | **Patients with BM**  **(n=86)** | **Patients without BM (n=158)** | ***P*-value** |
| --- | --- | --- | --- | --- |
| **Initial characteristics** | | | | |
| *Tumor biology group, n (%)*  HER2+ HR+  HER2+ HR-  HER2- HR+  Triple negative | 61 (25.0)  58 (23.8)  62 (25.4)  63 (25.8) | 22 (25.6)  23 (26.7)  20 (23.3)  21 (24.4) | 39 (24.7)  35 (22.2)  42 (26.6)  42 (26.6) | 0.836 |
| *ER status, n (%)*  Negative  Positive  Missing | 127 (52.9)  113 (47.1)  4 | 48 (55.8)  38 (44.2)  0 | 79 (51.3)  75 (48.7)  4 | 0.502 |
| *PR status, n (%)*  Negative  Positive  Missing | 182 (74.9)  61 (25.1)  1 | 65 (75.6)  21 (24.4)  0 | 117 (74.5)  40 (25.5)  1 | 0.855 |
| *HER2 status, n (%)*  Negative  Positive | 125 (51.2)  119 (48.8) | 41 (47.7)  45 (52.3) | 84 (53.2)  74 (46.8) | 0.412 |
| *Histological subtype, n (%)*  Ductal carcinoma  Other subtypes*  Missing | 201 (83.8)  39 (16.3)  4 | 73 (85.9)  12 (14.1)  1 | 128 (82.6)  27 (17.4)  3 | 0.507 |
| *Histological grade (SBR), n (%)*  1 or 2  3  Missing | 100 (45.5)  120 (54.5)  24 | 32 (41.0)  46 (59.0)  8 | 68 (47.9)  74 (52.1)  16 | 0.328 |
| *Inflammatory BC, n (%)*  No  Yes  Missing | 211 (90.6)  22 (9.4)  11 | 75 (89.3)  9 (10.7)  2 | 136 (91.3)  13 (8.7)  9 | 0.618 |
| *Metastatic status at BC diagnosis, n (%)*  M0  M1  Mx | 169 (71.0)  69 (29.0)  6 | 59 (68.6)  27 (31.4)  0 | 110 (72.4)  42 (27.6)  6 | 0.539 |
| *Adjuvant or néoadjuvant CT, n (%)*  No  Yes | 105 (43.0)  139 (57.0) | 40 (46.5)  46 (53.5) | 65 (41.1)  93 (58.9) | 0.418 |
| **Patients’ characteristics at the time of the serum sample** | | | | |
| *Median age in years (range)* | 58.3 (26.4 - 87.2) | 57.6 (26.4 – 79.6) | 58.4 (29.6 – 87.2) | 0.682 |
| *Age group, n (%)*  < 50  50 to 70  > 70 | 76 (31.3)  130 (53.3)  38 (15.6) | 28 (32.6)  47 (54.7)  11 (12.8) | 48 (30.4)  83 (52.5)  27 (17.1) | 0.673 |
| *Median number of lines of CT (range)* | 1 (0 - 9) | 1 (0 – 8) | 1.5 (0 – 9) | 0.554 |
| *Number of lines of CT, n (%)*  0 line  1 or 2 line(s)  > 2 lines | 58 (23.8)  110 (45.1)  76 (31.1) | 18 (20.9)  39 (45.3)  29 (33.7) | 40 (25.3)  71 (44.9)  47 (29.7) | 0.692 |
| *Previous anti-HER2 treatment among HER2+ patients, n (%)*  No  Yes | *n=119*  20 (16.8)  99 (83.2) | *n=45*  9 (20.0)  36 (80.0) | *n=74*  11 (14.9)  63 (85.1) | 0.468 |
| *Metastatic-free interval, n (%)*  ≤ 24 months  > 24 months | 112 (45.9)  132 (54.1) | 37 (43.0)  49 (57.0) | 75 (47.5)  83 (52.5) | 0.506 |
| *Number of metastatic sites, n (%)*  1-3  ≥4 | 101 (41.4)  143 (58.6) | 25 (29.1)  61 (70.9) | 76 (48.1)  82 (51.9) | 0.004 |
| *Location of metastatic sites, n (%)*  Bone and/or subcutaneous only  Visceral | 16 (6.6)  228 (93.4) | 0 (0.0)  86 (100.0) | 16 (10.1)  142 (89.9) | 0.009 |
| *Liver metastases, n (%)* | 148 (60.7) | 58 (67.4) | 90 (57.0) | 0.109 |
| *Bone metastases, n (%)* | 138 (56.6) | 51 (59.3) | 87 (55.1) | 0.523 |
| *Lung metastases, n (%)* | 121 (49.6) | 53 (61.6) | 68 (43.0) | 0.005 |
| *Lymph node metastases, n (%)* | 129 (52.9) | 55 (64.0) | 74 (46.8) | 0.010 |
| *Subcutaneous metastases, n (%)* | 42 (17.2) | 15 (17.4) | 27 (17.1) | 0.944 |
| *Pleural metastases, n (%)* | 52 (21.3) | 17 (19.8) | 35 (22.2) | 0.664 |
| *Metastases of other sites, n (%)* | 105 (43.0) | 42 (48.8) | 63 (39.9) | 0.177 |
| *ECOG status, n (%)*  0  1  2  3  Missing | 69 (30.5)  104 (46.0)  32 (14.2)  21 (9.3)  18 | 11 (14.1)  39 (50.0)  14 (17.9)  14 (17.9)  8 | 58 (39.2)  65 (43.9)  18 (12.2)  7 (4.7)  10 | <0.001 |
| *Anemia, n (%)*  No  Yes  Missing | 36 (25.4)  106 (74.6)  102 | 14 (21.9)  50 (78.1)  22 | 22 (28.2)  56 (71.8)  80 | 0.388 |
| *Leucopenia, n (%)*  No  Yes  Missing | 113 (81.9)  25 (18.1)  106 | 54 (84.4)  10 (15.6)  22 | 59 (79.7)  15 (20.3)  84 | 0.480 |
| *Neutropenia, n (%)*  No  Yes  Missing | 124 (88.6)  16 (11.4)  104 | 60 (93.8)  4 (6.3)  22 | 64 (84.2)  12 (15.8)  82 | <0.001 |
| *Lymphopenia, n (%)*  No  Yes  Missing | 79 (59.4)  54 (40.6)  111 | 33 (51.6)  31 (48.4)  22 | 46 (66.7)  23 (33.3)  89 | 0.181 |
| *Thrombopenia, n (%)*  No  Yes  Missing | 123 (87.9)  17 (12.1)  104 | 55 (85.9)  9 (14.1)  22 | 68 (89.5)  8 (10.5)  82 | <0.001 |
| *Elevated LDH, n (%)*  No  Yes  Missing | 48 (55.2)  39 (44.8)  157 | 30 (57.7)  22 (42.3)  34 | 18 (51.4)  17 (48.6)  123 | 0.056 |
| *Elevated serum CEA, n (%)*  No  Yes  Missing | 150 (66.4)  76 (33.6)  18 | 42 (51.9)  39 (48.1)  5 | 108 (74.5)  37 (25.5)  13 | 0.574 |
| *Elevated serum CA 15-3, n (%)*  No  Yes  Missing | 90 (39.8)  136 (60.2)  18 | 30 (37.0)  51 (63.0)  5 | 60 (41.4)  85 (58.6)  13 | <0.001 |
| *Elevated serum Tau (cut-off 3.17 pg/mL)*  No  Yes | 187 (76.6)  57 (23.4) | 57 (66.3)  29 (33.7) | 130 (82.3)  28 (17.7) | 0.002 |
| *Elevated serum HER2 ECD (cut-off 12.7 ng/mL)*  No  Yes | 104 (42.6)  140 (57.4) | 18 (20.9)  68 (79.1) | 86 (54.4)  72 (45.6) | <0.001 |
| *Elevated serum MMP-9 (cut-off 245 ng/mL)*  No  Yes | 74 (30.3)  170 (69.7) | 15 (17.4)  71 (82.6) | 59 (37.3)  99 (62.7) | <0.001 |
| *Low protein level, n (%)*  No  Yes  Missing | 78 (77.2)  23 (22.8)  143 | 48 (80.0)  12 (20.0)  26 | 30 (73.2)  11 (26.8)  117 | <0.001 |
| *Low albumin level, n (%)*  No  Yes  Missing | 130 (85.0)  23 (15.0)  91 | 61 (88.4)  8 (11.6)  17 | 69 (82.1)  15 (17.9)  74 | <0.001 |
| **Follow-up** | | | | |
| *Status as last follow-up, n (%)*  Alive  Dead | 74 (30.3)  170 (69.7) | 8 (9.3)  78 (90.7) | 66 (41.8)  92 (58.2) | <0.001 |
| *Cause of death, n (%)*  Oncological disease  Non-oncological  Toxic  Unknown | *(170 deceased)*  150 (88.2)  4 (2.4)  4 (2.4)  12 (7.1) | 65 (83.3)  3 (3.8)  2 (2.6)  8 (10.3) | 85 (92.4)  1 (1.1)  2 (2.2)  4 (4.3) | <0.001 |

* lobular carcinoma (n=21), mucinous carcinoma (n=1), papillary carcinoma (n=3), medullary carcinoma (n=1), mixed ductal and lobular carcinoma (n=12), other histological subtypes (n=4)

Abbreviations: BC: breast cancer; MBC: metastatic BC; BM: brain metastases; ER: estrogen-receptors; PR: progesterone-receptors; SBR: Scarf, Bloom and Richardson; BC: breast cancer; CT: chemotherapy; LDH: Lactate Dehydrogenase; CEA: Carcinoembryonic Antigen; CA 15-3: Cancer Antigen 15-3; HER2-ECD: HER2 extra-cellular domain; NSE: Neuron Specific Enolase; MMP-9: Matrix Metalloproteinase 9

**Supplementary Table 2**. Characteristics of brain metastases (n=86).

| **Characteristics** |  |
| --- | --- |
| *Brain as the first metastatic site, n (%)*  No  Yes | 71 (82.6)  15 (17.4) |
| *Prognostic score: Modified Breast-GPA, n (%)*  3.5-4  2.5-3  1.5-2  0-1  Not available | 7 (9.0)  30 (38.5)  23 (29.5)  18 (23.1)  8 |
| *Number of BM, n (%)*  1  2 or 3  4+ | 18 (20.9)  16 (18.6)  52 (60.5) |
| *Median BM volume, in cm^3^ (range)* | 7.1 (0.1 - 55.3) |
| *Mean BM volume, in cm^3^ (SD)* | 10.9 (12.27) |
| *BM localization, n (%)*  Supratentorial  Infratentorial  Both | 18 (20.9)  13 (15.1)  55 (64.0) |
| *Symptomatic BM, n (%)*  No  Yes | 30 (34.9)  56 (65.1) |
| *Intracranial hypertension symptoms, n (%)*  No  Yes | 47 (54.7)  39 (45.3) |
| *Neurological deficit, n (%)*  No  Yes | 48 (55.8)  38 (44.2) |
| *Epilepsy, n (%)*  No  Yes | 79 (91.9)  7 (8.1) |
| *Altered vigilance, n (%)*  No  Yes | 82 (95.3)  4 (4.7) |

Abbreviations: BM: brain metastases

**Supplementary Table 3**. Univariate analysis of OS (A) in the whole MBC population (n=244) and (B) in the BM population (n=86): additional results.

|  | **Whole MBC population (n=244)** | | **BM population (n=86)** | |
| --- | --- | --- | --- | --- |
| **Parameter** | **Median OS in months (95 CI%)** | ***P*-value** | **Median OS in months (95 CI%)** | ***P*-value** |
| **Initial characteristics** | | | | |
| *ER status*  Negative  Positive | 12.5 (10.0 – 19.1)  17.6 (12.4 – 27.8) | 0.104 | 6.8 (4.0 – 10.0)  11.6 (8.1 – 17.6) | 0.139 |
| *Histological subtype*  Ductal carcinoma  All other subtypes | 15.2 (11.6 – 20.6)  19.4 (10.1 – 35.6) | 0.314 | 9.8 (5.6 – 10.8)  8.4 (1.2 – 20.1) | 0.595 |
| *Inflammatory BC*  No  Yes | 15.1 (11.6 – 20.2)  20.9 (6.8 – NC) | 0.527 | 9.7 (5.3 – 10.4)  11.6 (1.5 – 20.9) | 0.797 |
| **Characteristics at the time of the serum sample** | | | | |
| *Age group*  < 50  50 to 70  > 70 | 15.0 (10.0 – 22.5)  16.3 (11.6 – 20.7)  20.2 (9.7 – 51.4) | 0.666 | 10.0 (6.4 – 14.4)  8.4 (3.8 – 10.8)  9.7 (1.0 – 20.2) | 0.864 |
| *Previous anti-HER2 treatment*  *(in HER2+ patients)*  No  Yes | NC  28.7 (19.2 – 33.1) | 0.266 | 19.1 (3.3 – NC)  10.0 (4.6 – 15.5) | 0.180 |
| *Metastatic-free interval*  ≤ 24 months  > 24 months | 15.0 (11.1 – 20.7)  16.2 (11.6 – 22.5) | 0.793 | 10.3 (4.7 – 19.1)  9.7 (4.7 – 10.8) | 0.323 |
| *Bone metastases*  Absent  Present | 17.6 (10.4 – 30.7)  15.2 (12.4 – 20.1) | 0.769 | 8.1 (4.6 – 10.4)  10.0 (4.7 – 13.6) | 0.835 |
| *Lung metastases*  Absent  Present | 20.7 (14.8 – 30.4)  12.4 (10.1 – 16.8) | 0.114 | 10.4 (4.6 – 17.6)  9.7 (4.7 – 10.4) | 0.457 |
| *Lymph node metastases*  Absent  Present | 19.4 (12.4 – 27.8)  13.6 (10.5 – 19.1) | 0.249 | 10.0 (4.6 – 12.4)  9.7 (4.7 – 12.4) | 0.990 |
| *Pleural metastases*  Absent  Present | 16.5 (12.5 – 20.7)  11.1 (4.6 – 28.7) | 0.059 | 10.0 (8.1 – 12.1)  2.8 (1.2 – 17.6) | 0.645 |
| *Anemia*  No  Yes | 15.5 (7.5 – 22.6)  10.3 (8.1 – 11.7) | 0.100 | 4.7 (1.2 – 15.5)  8.5 (4.0 – 10.4) | 0.499 |
| *Leucopenia*  No  Yes | 10.3 (8.1 – 12.4)  10.8 (6.4 – 31.6) | 0.329 | 8.1 (3.8 – 10.3)  8.1 (1.0 – 10.8) | 0.619 |
| *Neutropenia*  No  Yes | 10.6 (8.5 – 12.4)  10.4 (5.2 – NC) | 0.860 | 8.1 (4.0 – 10.3)  6.4 (1.0 – NC) | 0.871 |
| *Lymphopenia*  No  Yes | 11.1 (8.5 – 15.5)  8.4 (4.6 – 10.6) | 0.342 | 10.0 (4.7 – 13.6)  4.7 (2.2 – 9.8) | 0.491 |
| *Thrombopenia*  No  Yes | 10.6 (8.4 – 13.6)  10.1 (2.6 – 20.6) | 0.784 | 8.4 (3.8 – 10.3)  8.1 (0.7 – NC) | 0.146 |
| *Serum protein level*  Normal  Low | 9.7 (4.7 – 10.8)  8.1 (2.3 – 13.6) | 0.875 | 8.5 (4.2 – 10.8)  4.0 (1.2 – 10.3) | 0.069 |
| *Serum LDH*  Normal  Elevated | 10.3 (4.6 – 13.7)  4.7 (1.9 – 9.8) | 0.243 | 6.8 (3.8 – 10.8)  4.7 (1.4 – 10.4) | 0.660 |

Abbreviations: ER: estrogen-receptors; BC: breast cancer; CT: chemotherapy; LDH: Lactate Dehydrogenase; NC: not calculated
